# Effects of Cardiac Rehabilitation Training in Patients with Heart Failure Based on Traditional Chinese Exercise: A Systematic Review and Meta-Analysis

**DOI:** 10.1155/2021/1068623

**Published:** 2021-11-15

**Authors:** Fang Yao, Yang Zhang, Xiaohong Kuang, Qi Zhou, Lihua Huang, Jiazhu Peng, Kun Hou, Shizheng Du

**Affiliations:** ^1^Department of Nursing, Zhangjiagang TCM Hospital Affiliated to Nanjing University of Chinese Medicine, Suzhou, Jiangsu, China; ^2^School of Nursing, Nanjing University of Traditional Chinese Medicine, Nanjing, Jiangsu, China

## Abstract

**Methods:**

PubMed, Embase, Cochrane Library, and three Chinese databases, namely, China National Knowledge Network, Wanfang, and China Biomedical Network, were searched from the time of their inceptions through August, 2021. We retrieved the studies on the application of TCE-based cardiac rehabilitation in patients with HF. Based on the standard evaluation methods of Cochrane Reviewer's Handbook 5.1.0, two authors independently assessed the risk of bias and evaluated the methodological quality of the studies included. The RevMan 5.3 software was used for further meta-analysis. Additionally, the GRADEpro GDT web version was used to assess the quality of the evidence in these studies.

**Results:**

Nine randomized controlled trials involving 721 patients were included in this analysis. The meta-analysis revealed that the TCE (experimental group) effectively improved the patient's motor function and endurance compared to walking or other activities (control group) (mean difference, MD = 68.23, 95% CI [54.55, 81.91]; *P* < 0.00001). From each subgroup analysis, the exercising ability of the experimental group was higher than that of the control group. The quality of life's score in the experimental group was lower than that of the control group (MD = −9.51, 95%CI [−17.84, −1.18]; *P*=0.03). The plasma B-type natriuretic peptide content in the experimental group was lower than that in the control group (MD = −59.77, 95%CI [−82.85, −36.7]; *P* < 0.00001). The number of hospitalizations (MD = −0.83, 95%CI [−0.98, −0.68]; *P* < 0.00001) and hospital costs in the experimental group (MD = −1.6, 95%CI [−1.89, −1.31]; *P* < 0.00001) were lower than those in the control group. However, no significant differences were observed in the left ventricular ejection fraction and maximal oxygen consumption between the two groups (MD = 1.38, 95%CI [−3.08, 5.84] and *P*=0.54; MD = −0.04, 95%CI [−1.62, 1.54] and *P*=0.96, respectively). From the current analysis, TCE can be considered a relatively safe exercise method. According to the GRADE evaluation results on the evidence level, the studies included were of moderate quality, low quality, or very low quality.

**Conclusions:**

Our systematic review showed that TCE had potential benefits in improving patients' cardiac function, motor function, and quality of life. Therefore, TCE might be an effective adjuvant therapy in patients with HF. However, given the inclusion of the low-quality elucidations, further rigorous studies are urgently needed to confirm these results.

## 1. Introduction

Heart failure (HF) is mainly manifested by breathing difficulties during daily activities, generalized weakness, and liquid retention in the feet, legs, ankles, or stomach; it is a consequence of several cardiovascular diseases [1]. Considering the high morbidity, fatality rate, and rehospitalization rate, HF not only causes serious damage to the physical and psychological health and the living quality of the patients but also results in a serious economic burden to the families and society [2–4]. The prevention, treatment, and management of HF have become challenges to present and future research [5]. Based on the present studies [6, 7], specific exercise training and cardiac rehabilitation are important means to improve cardiac functions in patients with HF. This training constitutes a comprehensive medical treatment that integrates drug prescription, exercise plan, psychological scheme, diet plan, and risk factor control (including smoking cessation). Such an integrated approach is conducive to disease management and rehabilitation in patients with cardiovascular diseases at different stages. As a core therapy, cardiac rehabilitation and exercise rehabilitation have been proven safe and effective [8, 9], and this blended approach has advantages in improving cardiac function, strengthening exercise tolerance, and improving the quality of life in patients with HF. Chinese guidelines for the diagnosis and treatment of heart failure 2018 included exercise rehabilitation (aerobic exercises) as a recommended intervention approach for chronic HF management [10].

Traditional Chinese exercise (TCE) is practiced as a widespread therapy for exercise rehabilitation; the people practicing these exercises can benefit from its long-term clinical effect [11]. The TCE generally includes Taiji, Baduanjin, Wuqinxi, Yijinjing, other life cultivation and health preservation techniques, and other relevant practices [12]. According to TCE theories, this therapy not only requires respondents to perform physical exercises but also emphasizes the accommodation of psychology. The TCE adjusts the whole body's motion of “qi,” promoting blood circulation by constantly stimulating the acupoints and meridians, which can enhance the cardiac physiological function and promote cardiac disease recovery [13]. At the same time, TCE has the advantages of being simple and easy to learn with unlimited space and time and good compliance compared to the modern exercise rehabilitation regimen. Moreover, it can also meet the daily exercise needs in patients with HF. An earlier systematic review [14] confirmed that Taiji was an effective cardiac rehabilitation practice for patients with HF. Chen et al. [15] reported that Taiji and Qigong practices were promising rehabilitation therapies; however, some of their conclusions were based on a large heterogeneity, which was not confirmative. Therefore, the effects of TCE on heart failure remain unclear. Considering the growing number of randomized control trials on the TCE-based cardiac rehabilitation training used in HF patients, we conducted a systematic review and meta-analysis on the applications of TCE-based cardiac rehabilitation training in patients with HF. The research questions were as follows: (1) does the TCE-based cardiac rehabilitation training have a better clinical effect on the HF when compared to aerobic exercises or daily activities? (2) How are the evidence levels of relevant studies' results?

## 2. Data and Methods

### 2.1. Inclusion Criteria

#### 2.1.1. Populations

Patients diagnosed with heart failure [16] and in a stable phase of the disease with no acute manifestations without considering the ejection fraction heart failure measurements were included in the study.

#### 2.1.2. Intervention Group

1. Cardiac rehabilitation care: patients who were taking diuretics, vascular dilators, and digitalis correctly and undergoing disease observation, oxygen uptake monitoring, diet nursing, and mental nursing promptly were included in this analysis
2. Practicing TCE alone or in combination with Taiji, Baduanjin, Yijinjing, Wuqinxi, and Liuzijue was considered for this analysis
3. Intervention time of ≥3 months was included in this study

#### 2.1.3. Control Group

1. Cardiac rehabilitation care: the patients who were taking diuretics, vascular dilators, and digitalis accurately and undergoing disease observation, oxygen uptake monitoring, diet nursing, and mental nursing promptly were considered for this meta-analysis
2. Performing aerobic exercises or maintaining daily activities with the conventional medication and guidance of health education was considered for this analysis

#### 2.1.4. Research Type

Randomized controlled trial (RCT) studies analyzing the effects of traditional Chinese exercise regimens in patients with HF and publicly available were included.

#### 2.1.5. Outcome Indexes

 
*Primary Outcome Indexes.* The walking ability using a six-minute walk test (6MWT) and the quality of the patient's life using the Minnesota Living with Heart Failure Questionnaire (MLHFQ) were used
 
*Secondary Outcome Indexes.* Left ventricular ejection fraction (LVEF), B-type natriuretic peptide (BNP), maximal oxygen consumption (VO_2_max), and safety evaluation were used as secondary outcome variables

### 2.2. Exclusion Criteria

Exclusion criteria were defined as follows: (1) the patients with disrupted mobility, restricted independent body movement, and the structural valvular disease or chronic obstructive pulmonary disease were excluded; (2) the patients with the cognitive disorder or severe depression were omitted from the analysis; (3) repeated publications were removed; and (4) case reports, review articles, and animal experiments were removed from the analysis.

### 2.3. Literature Resources and Search Strategies

This review was performed according to the Preferred Reporting Items for Systematic Review and Meta-Analysis (PRISMA) statement. Chinese and foreign databases such as CNKI, Wanfang, Chinese Biological Medicine Network, PubMed, Cochrane Library, and Embase were used for the comprehensive online search. A combination of subject words and free words was used. The following search terms were used for the TCE: *Taiji* OR *Tai chi* OR *Taijiquan* OR *Qigong* OR *Baduanjin* OR *Baduanjin exercise* OR *Eight Section Brocades* OR *Wuqinxi* OR *Five-animal exercises* OR *Liuzijue* OR *Yijinjing* OR *traditional exercises OR traditional exercise therapy*. The following search terms were used for the HF: *heart* OR *heart failure* OR *chronic cardiac insufficiency* OR *cardiac failure* OR *cardiac rehabilitation.* Moreover, the references included in the studies retrieved primarily were searched again from the inception through August, 2021.

### 2.4. Literature Screening and Data Extraction

Two evaluators (LHH and JZP) screened the studies and extracted the data independently based on the inclusion and exclusion criteria. The titles and abstracts were examined first, and the unrelated articles were removed. Subsequently, the full texts were scrutinized, and more irrelevant records were removed. Then, a data extraction table was formatted. If any disagreement was present, the two evaluators negotiated to solve the disagreement. If an agreement could not be reached after the negotiation, a third researcher (QZ) was consulted to solve the disagreement. The data extraction contents were collated into a table following the Consort Statement and corresponding standards of the traditional medicine reports [17]; the contents mainly included (1) general data: titles of the studies, the first author, and the year of publishing; (2) research features: subjects involved, sample size, intervention measures, and the frequency of the intervention; and (3) primary and secondary outcome indexes.

### 2.5. Evaluation of the Risk of Bias in the Studies Included

This review followed the standards of Cochrane Reviewer's Handbook version 5.1.0, and the quality of the included methodologies was assessed [18]. The standards mainly included the following conditions: (1) whether the random method is appropriate; (2) whether it is hidden by the allocation plans; (3) whether the blind treatment is applied to the patient and the researchers; (4) whether the blinding is applied to the evaluators of outcome measures; (5) whether the bias is caused by the missing data; (6) whether the bias is caused by selective information; (7) whether other types of bias exist. On this basis, each index was assessed by the low and high bias risks or ambiguity (relative information missing or inexplicit bias). Two literature evaluators (KH and YZ) were invited for the independent determination, and the result data were checked repeatedly by crossing out. For comparing the results, a third researcher (QZ) was further referred to any disagreement, and the secondary analysis and assessment were performed to reach the final decision.

### 2.6. GRADE Classification

The GRADEpro GDT web page was employed to evaluate the evidence for the quality of research outcome used in the analysis. The evaluation standards included the limitations of the studies; inconsistencies of the results; and indirectness, precision, and publishing biases. Four recommendation grades were involved: “high,” “moderate,” “low,” and “very low.”

### 2.7. Data Analysis

Statistical analysis of data was carried out using the RevMan 5.3 software. Firstly, the existence of the clinical heterogeneities among different studies was assessed by the *χ*^2^-test and *I*^2^-judgment. If a small heterogeneity was present (*P* > 0.1, *I*^2^ < 50%), the fixed effects model (FEM) was used for the analysis. If clinical heterogeneity was present (*P* ≤ 0.1, *I*^2^ ≥ 50%), the heterogeneity source was discussed and the subgroup analysis was performed under the necessary conditions [19]. For various test results of the evident clinical heterogeneity, a random-effects model (REM) was employed for the analysis. The continuous variables used the mean difference (MD) or weight mean difference (WMD) as the effect analysis. The odds risk was determined for the classified variables, and a 95% confidence interval (CI) was calculated by the effect analysis. The test standard was *α* = 0.05. A funnel graph was drawn to evaluate the potential publication bias.

## 3. Results

### 3.1. Literature Review

**Figure 1:**
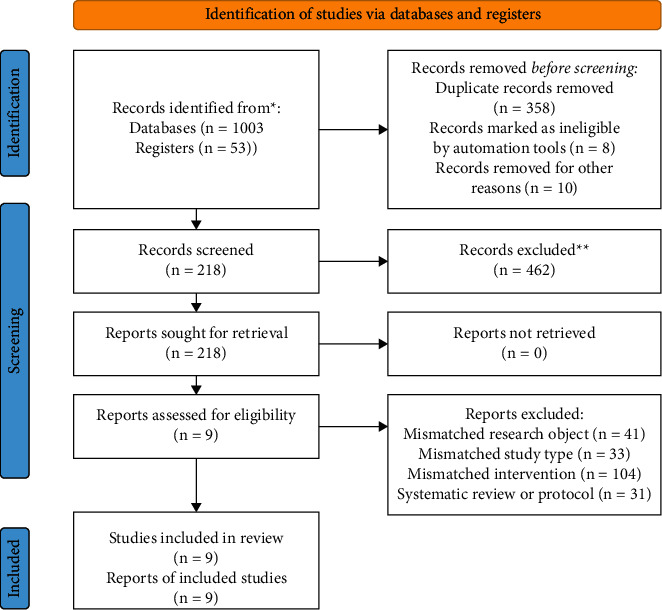
Flow diagram of the selection of studies.

A total of 1056 publications, including 149 publications in English and 907 publications in Chinese, were obtained through the preliminary database search and other means. We also checked the https://ClinicalTrials.gov database, and four studies that are currently recruiting and analyzing were unable to be incorporated in this meta-analysis. After reading the titles and abstracts, the studies that were published repeatedly and showed disagreement with their topics were excluded. A total of 218 publications required full-text reading. Finally, nine publications were included in the study (Figure 1).

### 3.2. Basic Features of the Studies Included

Nine randomized control studies [20–28] were included in this analysis. Three studies [21–23] were performed in America, and one [20] was conducted in Taiwan, China. The remaining five studies [24–28] were performed in Mainland China. These studies consisted of 721 patients, including 359 in the experimental groups and 362 in the control groups (Table 1).

### 3.3. Assessing the Risk of Bias in the Studies Included

**Figure 2:**
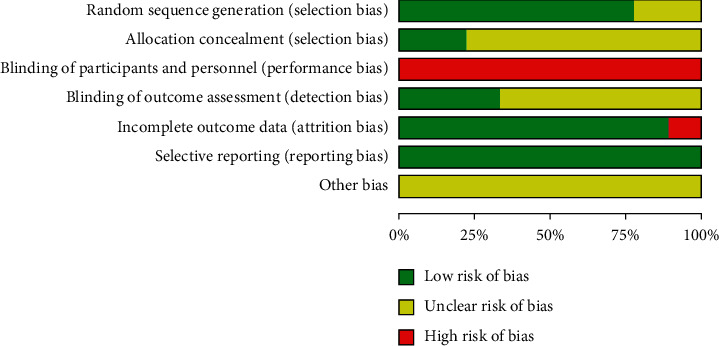
Bar graph showing the risk of bias: the authors' judgments on the risk of each category of the bias that are presented as percentages across all included studies.

**Figure 3:**
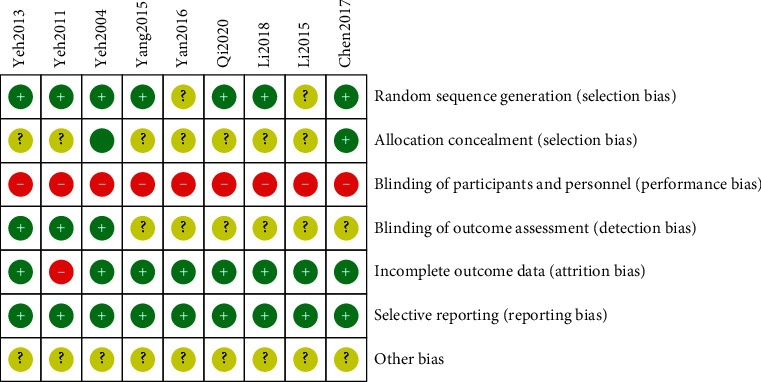
Risk of the bias summary.

Figures 2 and 3 show the specific evaluation of the results and risk bias assessment.

### 3.4. Meta-Analysis Results

#### 3.4.1. Main Outcome Indexes

**Figure 4:**
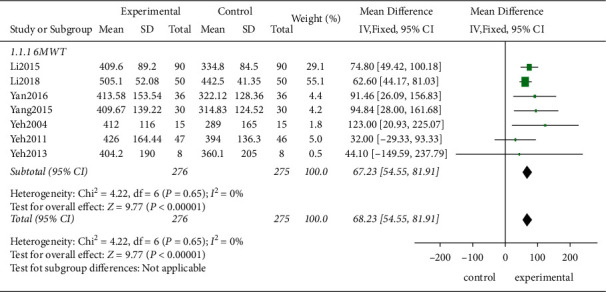
Meta-analysis of the 6MWT.

*(1) The 6MWT*. In the nine studies included, seven studies evaluated the changes in the exercise tolerance before and after the treatment of patients using the 6MWT. According to the *χ*^2^-test, no statistical heterogeneity existed among the studies (*P*=0.65, *I*^2^ = 0%). Therefore, the FEM was employed. The results showed that the traditional exercise rehabilitation prolonged the distance of the 6MWT significantly compared to the control group showing a statistically significant difference (MD = 68.23, 95% CI [54.55, 81.91], *P* < 0.00001; Figure 4).

*(2) Subgroup Analysis*. Although no statistical heterogeneity was observed among the studies, certain clinical heterogeneities might have been present due to the variation in the intervention measures and the HF characteristics. Therefore, the influences of the intervention period, intervention measures, and the HF type on the 6MWT in the three subgroups were compared.

**Figure 5:**
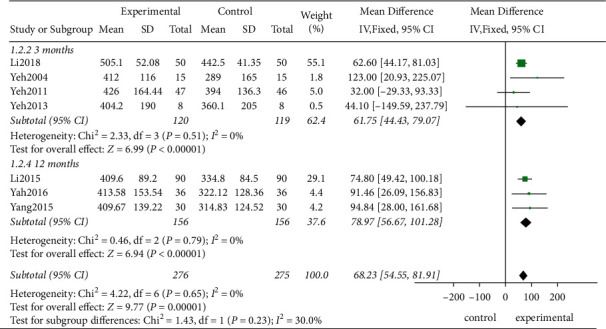
The subgroup analysis for different treatment periods.

**Figure 6:**
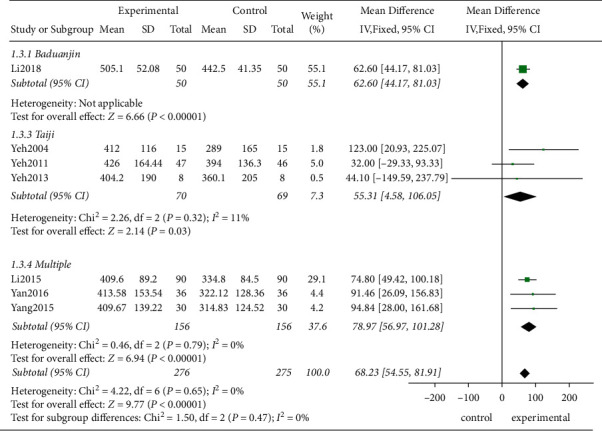
The subgroup analysis for different interventions.

**Figure 7:**
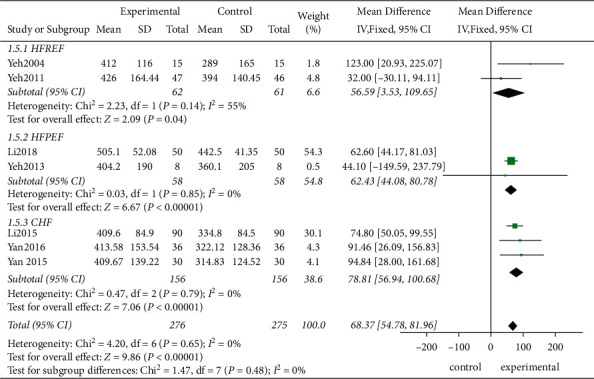
The subgroup analysis for different types of HF.

1. Firstly, the studies demonstrated that after the intervention for 3 and 12 months, the experimental group achieved better therapeutic effects than the control group, and a relatively long treatment period (MD = 78.97, 95% CI [56.67, 101.28]; *P* < 0.00001) showed a better intervention effect than a short treatment period (MD = 61.75, 95% CI [44.43, 79.07], *P* < 0.00001) (Figure 5)
2. Based on the different intervention measures, the combination of several traditional exercises and Taiji achieved better effect than the control groups showing statistically significant differences (MD = 78.97, 95% CI [56.67, 101.28]; *P* < 0.00001; MD = 55.31, 95% CI [4.58, 106.05]; *P*=0.03, respectively, Figure 6)
3. Finally, this study confirmed that TCE showed a beneficial effect for managing HF with the preserved or no-ejection fraction (MD = 68.37, 95% CI [54.78, 81.96]; *P* < 0.00001, Figure 7)

**Figure 8:**
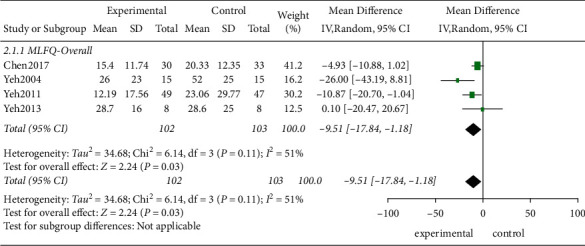
The meta-analysis for the MLHFQ scores.

*(3) The MLHFQ*. Four studies evaluated the quality of life in 205 patients using the MLHFQ score. According to the *χ*^2^-test, heterogeneity existed among the studies (*P*=0.11, *I*^2^ = 51%), and the REM was applied. The results demonstrated that the TCE improved the quality of life of the patients effectively compared to the control group, and the difference showed a statistical significance (MD = −9.51, 95% CI [−17.84, −1.18]; *P*=0.03; Figure 8).

#### 3.4.2. Secondary Outcome Indexes

**Figure 9:**
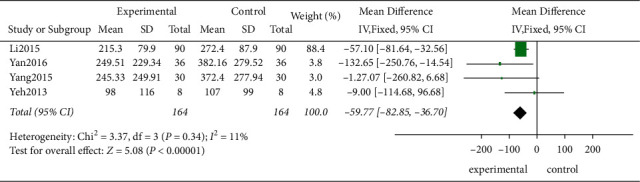
The meta-analysis of the BNP.

*(1) The BNP*. Four studies used the BNP to evaluate the HF degree in 328 patients. According to the *χ*^2^-test, no statistical heterogeneity was observed among these studies (*P*=0.34, *I*^2^ = 11%), and the FEM was applied. The results indicated that the TCE decreased the plasma BNP level effectively compared to the control group showing a statistically significant difference (MD = −59.77, 95% CI [−82.85, −36.7]; *P* < 0.00001, Figure 9).

**Figure 10:**
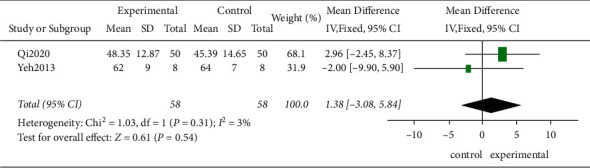
The meta-analysis of the LVEF.

*(2) The LVEF*. Two studies tested the LVEF values in 116 patients. According to the *χ*^2^-test, no statistical heterogeneity existed among these studies (*P*=0.31, *I*^2^ = 3%), and the FEM was employed. According to the results, no evidence suggested that the TCE improved the LVEF effectively compared to the control group, showing no statistically significant difference (MD = 1.38, 95%CI [−3.08, 5.84]; *P*=0.54; Figure 10).

**Figure 11:**
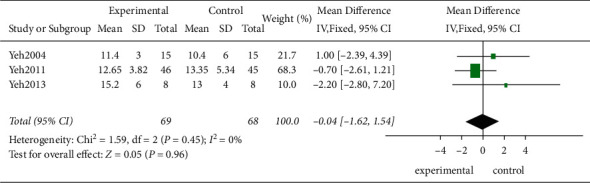
The meta-analysis for the VO_2_max.

*(3) The VO_2_max*. Three studies evaluated the maximum oxygen uptake in 137 patients using the variable VO_2_max. According to the *χ*-test, no statistical heterogeneity existed among these studies (*P*=0.45, *I*^2^ = 0%), and the FEM was used. The results from the model revealed that the traditional exercise rehabilitation did not increase the maximum oxygen uptake in the patients effectively compared to the control group showing no statistically significant difference (MD = −0.04, 95% CI [−1.62, 1.54]; *P*=0.96; Figure 11).

**Figure 12:**
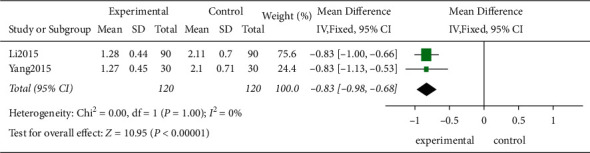
The meta-analysis for the number of hospitalizations.

*(4) The Number of Hospitalizations*. Two studies evaluated the number of hospitalizations in 240 patients. According to the *χ*^2^-test, no statistical heterogeneity was identified among these studies (*P*=1.00, *I*^2^ = 0%), and the FEM was used for the analysis. As shown by the results, the TCE decreased the number of hospitalizations compared with the control group showing a statistically significant difference (MD = −0.83, 95% CI [−0.98, −0.68]; *P* < 0.00001; Figure 12).

**Figure 13:**
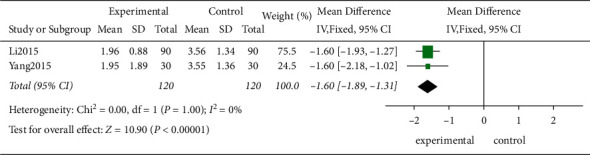
The meta-analysis of the hospital costs.

*(5) The Hospital Costs*. Two studies evaluated the hospital costs in 240 patients. According to the *χ*^2^-test, no statistical heterogeneity was present among these studies (*P*=1.00, *I*^2^ = 0%), and the FEM was used for further analysis. The results showed that the traditional exercise rehabilitation decreased the hospital costs of the patients compared to the control group showing a statistically significant difference (MD = −1.6, 95% CI [−1.89, −1.31]; *P* < 0.00001; Figure 13).

### 3.5. Evaluation of the GRADE Evidence Quality

Using the web version of the Cochrane collaboration network GRADEpro GDT, the evidence quality of these studies was evaluated. According to the evaluation, one study result showed a “moderate quality,” four studies revealed a “low quality,” and two studies exhibited a “very low quality” as shown in Table 2.

### 3.6. Safety Evaluation

Among the nine included studies, five studies failed to mention the occurrence of the adverse events, and four publications reported no serious adverse events at the end of the exercise period. A meta-analysis on the safety of these studies cannot be performed, but the TCE is considered safe from the description.

### 3.7. The Publication Biases

**Figure 14:**
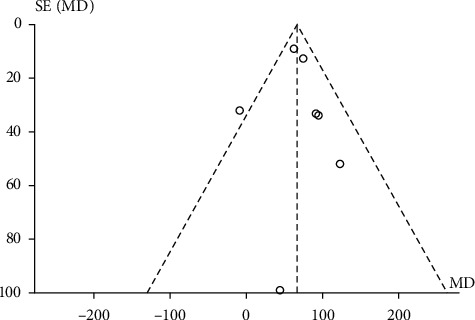
The funnel plot for the publication bias.

This study included nine randomized control trials in total including seven studies that evaluated the 6MWT. These seven studies were visualized using a funnel graph [29], showing publication bias to a certain extent (Figure 14).

## 4. Discussions

### 4.1. The Quality of the Methodologies in the Publications Analyzed

Several methodologies in the included studies showed poor quality, thus resulting in the high risk of bias. This outcome may influence the accuracy of this conclusion to a certain extent. In the included nine studies, seven publications explained the random grouping method comprehensively, and two studies had random grouping; however, the random grouping method was not explained thoroughly. Two studies had the hidden allocation. One literature had the survey data missing, and no intentional analysis was performed. Two studies implemented a blind approach to the outcome evaluators. Given the difficulty of keeping patients and facilitators blind to the TCE, none of the nine studies adopted the blinded treatment approach to the patients and executors, which led to certain risks.

### 4.2. Effects of Cardiac Rehabilitation Therapy Based on the TCE on Motor Function of the Patients

Kinetotherapy is the core therapy in cardiac rehabilitation; it has become one of the methods used to control cardiac failure in recent years. As an effective objective index that evaluates exercise tolerance and judges the prognosis of the patients with HF, 6WMT is considered noninvasive, simple, and safe. It has high repeatability and is easily accepted by the patients [30, 31]. Currently, 6WMT is widely employed in various diseases, such as HF and chronic lung obstructive pulmonary disease [32]. A high score from the 6WMT indicates a high exercise tolerance in the patients. According to the meta-analysis results, TCE prolonged the 6 min walking distance compared to the control group. According to the subgroup analysis, the patients showed an improved exercise tolerance with the prolonged intervention time and combined exercise methods. HF exhibits the symptoms of edema, palpitation, and gasping. Based on the qi deficiency, HF shows cardiac weakness, circulatory disorder, and respiratory tract obstruction with phlegm, resulting in blood stagnation and water retention, which further aggravates heart failure [33]. TCE is a unique combination of physical and psychological interventions that focus on postures, breathing processes, and meditation [34]. This exercise modulates qi deficiency by the flexion and extension of the joints, prostration of the body, activation of muscles and tendons, promotion of the blood circulation, and enhancing the organ functions [35], thus improving control of the body, enhancing limb movements, and promoting physical and psychological coordinated health. Another key benefit of the TCE is regular breathing by coordinated respirations with physical movement and self-imagination. This process can decrease the tension of cardiac amphotony and ventricular loads, relieve the activity burden, and strengthen the exercise tolerance in the patients [36]. According to recent studies [37], TCE can promote muscle fiber strength and improve muscle perfusion and metabolism, thus strengthening muscle strength and increasing exercise tolerance in patients with HF.

### 4.3. Effects of Cardiac Rehabilitation Based on TCE on the Quality of Life in Patients

As a result of fatigue, palpitation, and breathing difficulties, the patients with HF are restricted to the performance of daily activities and they easily develop negative emotions such as anxiety and depression. Consequently, they suffer from the disorder of the nervous system, and their cardiac functions further worsen, thus decreasing their quality of life. In addition, patients with HF easily have other complications [38], which can prolong the hospitalization time, thus negatively affecting the quality of life. BNP is a cardiac neurohormone excreted by the cardiomyocytes, and an excessive pressure load can stimulate the ventricle to secrete the BNP, whose level is positively related to the patient's cardiac functional classification. According to the results of this meta-analysis, TCE decreased the level of BNP effectively and was positively related to mitigating HF. The LVEF is positively related to myocardial contractility. In a study, when the myocardial contractility was strong, the LVEF was high, but this study lacked the evidence to prove that the TCE improved the LVEF and increased the cardiac ejection in the patients with HF. The VO_2_max was closely related to the effective ventilation and normal cardiac output [39], and in this study, the TCE could not improve the VO_2_max effectively compared to the control group. Moreover, TCE decreased the number of hospitalizations and hospital costs of patients. MLHFQ is sensitive and effective, and it is appropriate to evaluate the quality of life in patients with HF in China. A high score indicates the poor quality of life of patients. This study revealed that the TCE reduced the MLHFQ score of patients with HF and improved their quality of life, which agrees with a previous report by Pan et al. [40] As a unique nonpharmaceutical therapy, TCE can develop cardiovascular protection through multiple pathways and several target points to prevent the myocardial fibrosis, inhibit the myocardial degeneration, and improve the myocardial function in China [41, 42]. Based on the meridian qi and blood theory of the TCM, TCE requires gentle movements and regular and relaxed breathing, which can “motivate spirit and strengthen soul” and “rest the mind and stabilize the qi.” The combination of the active movements and breathing processes can enhance the joint and muscular activities systematically, improve the respiratory functions, and relieve the pressure. Additionally, previous studies [43, 44] proved that TCE also had antianxiety and antidepression effects. With the improvement of exercise tolerance, cardiac function and psychological state, reduced number of hospitalizations and hospital costs, decreased limitation over daily activities, and relief of the pressure and burden, the quality of life in the patients can improve.

### 4.4. Research Limitations and Suggestions

This analysis also had several limitations. (1) Although all nine studies used random grouping, two elucidations provided incomplete descriptions regarding random grouping. Two studies reported allocation concealment, and none of the nine studies adopted a blind approach to the patients. Some studies lacked reports about the objective outcome indicators such as blood pressure, heart rate, cholesterol level, and triglycerides content, which made the meta-analysis impossible to combine them for evaluation. (2) Some studies showed heterogeneity after being combined, which might have been caused by differences in the included studies specific to the intervention frequency, duration, types of the intervention measures, and the underlying diseases. (3) The argumentation strength of this study may have been influenced by the small sample size of the included studies, imperfect research outcome indexes and design, and biases to a certain degree. For specific indexes such as the LVEF, the number of hospitalizations, and the hospital costs, only two studies were available for the analysis, which may have led to the inadequate extrapolation of the results.

Based on the above limitations, the suggestions were made for the clinically relevant future studies. (1) Researchers should design a scientific and reasonable research design and use random grouping method, allocation hidden and blinded method to further improve the methodological quality of researches. The objective indicators should be used to increase the credibility of the results. (2) The TCE training program should be further standardized to have detailed explanations and standards for the training duration, training intensity, and frequency. (3) The patients should be followed up as long as possible to observe their long-term therapeutic effects, given the long-term benefit of TCE. (4) Multicenter, large-sample-sized, and high-quality clinical randomized control studies should be implemented.

## 5. Conclusions

This study aimed to discuss the influences of cardiac rehabilitation using TCE in patients with HF. This meta-analysis showed that TCE had beneficial effects on motor function, cardiac function, and the quality of life in patients with HF. Moreover, this form of therapy is beyond limitations because of its low-cost requirement. TCE may be an efficient substitute for exercise rehabilitation in patients with HF.

## Figures and Tables

**Table 1:** The characteristics of the nine studies retrieved from the literature search.

Study	LVEF	Year	Heart function class	Sample size		Intervention		Frequency (times/week)	Duration (min/each)	Treatment (month)	Outcome
				Experiment	Control	Experiment	Control				
Qi et al.	CHF	2020	II–III	50	50	CR care + Baduanjin	CR care + daily activities	No	According to bullseye heart rate	3	(2), (3)
Li et al.	HFPEF	2018	II–III	50	50	CR caret + Baduanjin	CR care + walking training	≥5	30	3	(1), (3)
Li et al.	CHF	2015	II–IV	90	90	CR care + Yijinjing + Wuqinxi + Liuzijue + Baduanjin	CR care + daily activities	7	No	12	(1), (4), (6), (7)
Yang et al.	CHF	2015	I–IV	30	30	CR care + Liuzijue + Baduanjin	CR care + daily activities	5–10	20–30	12	(1), (4), (6), (7)
Yan	CHF	2016	I–IV	36	36	CR care + Liuzijue + Baduanjin	CR care + daily activities	No	No	12	(1), (4)
Yeh et al.	HFREF	2004	I–IV	15	15	CR care + Taiji	CR care + daily activities	2	60	3	(1), (3), (5)
Yeh et al.	HFREF	2011	I–III	50	50	CR care + Taiji	CR care + daily activities	2	60	3	(1), (3), (5)
Yeh et al.	HFPEF	2013	I–III	8	8	CR care + Taiji	CR care + aerobic exercise	2	60	3	(1), (2), (3), (4), (5)
Chen et al.	CHF	2017	I–II	30	33	CR care + Baduanjin	CR care + daily activities	3	35	3	(3)

Note: (1) 6MWT; (2) LVEF; (3) MLHFQ; (4) BNP; (5) VO_2_max; (6) number of rehospitalization; (7) hospital costs.

**Table 2:** GRADE evidence quality results.

Certainty assessment							No. of patients		Certainty
No. of studies	Study design	Risk of bias	Inconsistency	Indirectness	Imprecision	Other considerations	TCE	Other activities	
*6MWT*									
7	Randomized trials	Serious^a^	Not serious	Not serious	Not serious	Publication bias strongly suspected^b^	276	275	⊕⊕⊕○ Moderate

*MLHFQ*									
4	Randomized trials	Serious^a^	Serious^c^	Not serious	Serious^d^	None	102	103	⊕○○○ Very low

*LEVF*									
2	Randomized trials	Serious^a^	Not serious	Not serious	Serious^d^	None	58	58	⊕⊕○○ Low

*BNP*									
4	Randomized trials	Serious^a^	Serious^c^	Not serious	Serious^d^	None	164	164	⊕○○○ Very low

*VO*_*2*_ *max*									
3	Randomized trials	Serious^a^	Not serious	Not serious	Serious^d^	None	69	68	⊕⊕○○ Low

*Number of rehospitalisation*									
2	Randomized trials	Serious^a^	Not serious	Not serious	Serious^d^	None	120	120	⊕⊕○○ Low

*Number of hospitalisations and costs*									
2	Randomized trials	Serious^a^	Not serious	Not serious	Serious^d^	None	120	120	⊕⊕○○ Low

CI: confidence interval; MD: mean difference. ^a^None of the included articles used blinding approach, and a high risk of bias was present; ^b^publication bias may be present; ^c^the heterogeneity was substantial (*I*^2^ ≥ 50%); ^d^the total number of participants in both groups was less than 400.
